# A fast weak motif-finding algorithm based on community detection in graphs

**DOI:** 10.1186/1471-2105-14-227

**Published:** 2013-07-17

**Authors:** Caiyan Jia, Matthew B Carson, Jian Yu

**Affiliations:** 1 School of Computer and Information Technology, Beijing Jiaotong University, Beijing 100044, China; 2 Department of Bioengineering/Bioinformatics, University of Illinois at Chicago, Chicago, IL 60612, USA

## Abstract

**Background:**

Identification of transcription factor binding sites (also called ‘motif discovery’) in DNA sequences is a basic step in understanding genetic regulation. Although many successful programs have been developed, the problem is far from being solved on account of diversity in gene expression/regulation and the low specificity of binding sites. State-of-the-art algorithms have their own constraints (e.g., high time or space complexity for finding long motifs, low precision in identification of weak motifs, or the OOPS constraint: one occurrence of the motif instance per sequence) which limit their scope of application.

**Results:**

In this paper, we present a novel and fast algorithm we call TFBSGroup. It is based on community detection from a graph and is used to discover long and weak (*l*,*d*) motifs under the ZOMOPS constraint (zero, one or multiple occurrence(s) of the motif instance(s) per sequence), where *l* is the length of a motif and *d* is the maximum number of mutations between a motif instance and the motif itself. Firstly, TFBSGroup transforms the (*l*, *d*) motif search in sequences to focus on the discovery of dense subgraphs within a graph. It identifies these subgraphs using a fast community detection method for obtaining coarse-grained candidate motifs. Next, it greedily refines these candidate motifs towards the true motif within their own communities. Empirical studies on synthetic (*l*, *d*) samples have shown that TFBSGroup is very efficient (e.g., it can find true (18, 6), (24, 8) motifs within 30 seconds). More importantly, the algorithm has succeeded in rapidly identifying motifs in a large data set of prokaryotic promoters generated from the *Escherichia coli* database RegulonDB. The algorithm has also accurately identified motifs in ChIP-seq data sets for 12 mouse transcription factors involved in ES cell pluripotency and self-renewal.

**Conclusions:**

Our novel heuristic algorithm, TFBSGroup, is able to quickly identify nearly exact matches for long and weak (*l*, *d*) motifs in DNA sequences under the ZOMOPS constraint. It is also capable of finding motifs in real applications. The source code for TFBSGroup can be obtained from http://bioinformatics.bioengr.uic.edu/TFBSGroup/.

## Background

Transcription factors play an irreplaceable role in the activation and repression of gene expression by binding to specific sites within promoter regions of target genes. Identification of transcription factor binding sites (TFBSs) is a basic task for elucidating the molecular mechanisms of transcriptional regulation. Although traditional footprinting assays can accurately identify the precise binding sites of any factor, this low-throughput method is highly technical and can analyze only a single small region (< 1 kb) at a time. With the development of high-throughput sequencing technologies, a number of experimental techniques such as ChIP-chip and ChIP-seq have been used to identify the location of transcription factor binding sites. However, these methods are unable to resolve DNA-protein interactions at base pair resolution [1]. *In silico* identification of over-represented DNA motifs from the promoters of co-regulated or homologous genes as well as ChIP-enriched regions plays a significant role in locating binding sites in a high-throughput and high-resolution manner.

Since a DNA motif is usually highly conserved or over-represented among DNA sequences, there are two main approaches to its representation: (1) represent a motif by its profile or position-specific scoring matrix (PSSM) [*n*_*j*,*k*_]_*l*×4_, which records the frequency of base *k* (*k*∈{*A*,*C*,*G*,*T*}) at position *j* (*j*={1,2,⋯,*l*}) for all aligned sites [2-4] or (2) characterize a motif as an *l*-length consensus string describing a motif with the most frequent nucleotide in each position of all aligned sites. According to these two TFBS models, existing motif-finding algorithms can be divided into two classes. The first includes algorithms that maximize a statistical or entropy-related score of a PSSM [*n*_*j*,*k*_]_*l*×4_. CONSENSUS [5], MEME [6], Gibbs Sampler [7], AlignACE [8], PROJECTION [9], and CRMD [10] belong to this group. These algorithms use optimization techniques from the fields of statistics and machine learning, including the greedy strategy [5], the Expectation-Maximization method [6], Gibbs sampling methods [7-9], and the clustering method [10]. These algorithms usually have a fast run time. Sometimes, however, they cannot converge to the global optimum, especially for short motifs with high levels of statistical noise or long motifs with a large search space. The second class of algorithms usually searches for (*l*, *d*) motifs based on the consensus model [11] by employing heuristic methods, but in some cases use optimal techniques. It is supposed that each sequence contains zero, one, or multiple motif instance(s) with up to *d* mutations within a true motif [12]. A large number of algorithms have been proposed to exactly or almost exactly extract (*l*, *d*) motifs from *N* input sequences with length *L*. SPELLER [13], WEEDER [14,15], MITRA-count [16], Voting [17], PMSprune [18], WINNOWER [11], iTriplet [19], VINE [20], Stemming [21], RecMotif [22], and sMCL-WMR [23] are included in this group. These algorithms usually have a high time complexity for long motifs. This limits their application, especially toward prokaryotic promoters [24,25]. In this study, we intend to offer a highly efficient algorithm for finding long and weak (*l*, *d*) motifs and to use this algorithm to identify TFBSs in prokaryotic data sets.

Motif-finding algorithms based on the consensus model can be further divided into two categories: pattern-driven and sample-driven approaches [16]. Using a pattern-driven approach, one tries to enumerate all possible 4^*l*^*l*-mer motifs with lexical order. When applying a sample-driven approach, all possible (*l*, *d*) motifs generated from the real *l*-mers of input sequences are often tested. SPELLER, WEEDER, and MITRA-count are pattern-driven approaches and Voting, PMPprune, WINNOWER, iTriple, VINE, Stemming, RecMotif, and sMCL-WMR are sample-driven. In general, pattern-driven approaches can automatically find (*l*, *d*) motifs in samples without being given the length *l*. However, the state-of-the-art sample-driven approaches are usually faster than the state-of-the-art pattern-driven approaches, and thus can be used to extract motifs with a larger *l* and *d*.

Recently, the sample-driven approach, which transforms the (*l*,*d*) motif search by extracting the maximum clique or *q*-cliques (*q*≤*N*) from an *N*-partite graph, has attracted much attention. In this graph, each vertex is an *l*-mer. There is an edge between two *l*-mers from two different sequences if the Hamming distance between these two *l*-mers is no more than 2*d*. This is because the Hamming distance between each instance of a motif and the motif itself is assumed to be at most *d*. Thus, all instances of a motif must form a maximum clique or *q*-cliques in the graph. This idea was first presented in WINNOWER, which utilizes an extendable mechanism to cut off spurious edges by extending *k*-cliques (*k*=2 or *k*=3) to larger (*k*+1)-cliques. However, the accuracy of WINNOWER cannot be guaranteed since there is strong background noise in many sequences and true edges may be pruned by its local extension mechanism. The VINE algorithm, with its rigorous pruning steps, was proposed to speed up and increase the precision rate of WINNOWER. Similarly, iTriplet was designed to randomly select two reference sequences and identify all triplets (3-cliques) in these as well as each of the remaining *N*−2 sequences. All discovered triplets along with their sequence information are then inserted into hash tables as candidate motifs. If a table has enough instances (e.g., at least *q*), the motif can be identified as ‘true’. RecMotif was created to extract *N*-cliques by using reference sequences as well. It takes the selected reference vertices from the first *x* (*x*={1,2,⋯,*N*}) reference sequences in order to select new reference vertices in the remaining sequences. As *x* is increased, the selection is continued (*x*←*x*+1) if new reference vertices can be selected from the remaining sequences to obtain *x*-cliques. Otherwise, the algorithm backtracks to the first *x*−1 reference vertices and finds a substitute. RecMotif has been shown to be very fast for some (*l*, *d*) cases (e.g., (15, 4), (18, 5) and (21, 6)). However, in tests performed by Sun et al. [22], it failed to find some weak motifs such as (15, 5), (18, 6), and (19, 7). Additionally, RecMotif operates under the OOPS constraint. During real applications, some sequences may not contain any instance of a true motif while some may contain multiple instances. With this work, we offer a more efficient algorithm for extracting long and weak (*l*, *d*) motifs from *N*-partite graphs using the more biologically-relevant ZOMOPS constraint.

During our research, we made the following observations: (1) there may be too many spurious edges in an *N*-partite graph to extract the *q*-cliques (*q*≤*N*) needed to identify a weak motif (e.g., (15, 4) or (18, 6)) and (2) if we construct the *N*-partite graph such that there is an edge between two *l*-mers from two different sequences if the Hamming distance between these two *l*-mers is no more than *x* (*d*≤*x*≤2*d*) instead of exactly 2*d*, the motif instances in the graph may form a dense subgraph instead of a clique. Based on this information, we present a new algorithm: TFBSGroup. It first extracts dense subgraphs, which are groups of candidate instances (i.e., TFBSs), by the fast community detection algorithm BGLL [26]. It then greedily refines these candidate motifs towards the true motif within their own communities. Our empirical study shows that TFBSGroup can quickly discover long and weak (*l*, *d*) motifs (e.g., (18, 6) and (24, 8) motifs within 30 seconds) in synthetical samples under the ZOMOPS constraint. More importantly, it is able to rapidly identify motifs in a large data set of prokaryotic promoters [25] generated from the *Escherichia coli* database RegulonDB [27]. It is also able to accurately discover motifs in 12 mouse transcription factor ChIP-seq data sets involved in ES cell pluripotency and self-renewal [28].

## Results

We first tested TFBSGroup on a series of synthetic (*l*, *d*) samples and compared it with iTriplet (source code: http://www.rci.rutgers.edu/ ∼gundersn/iTriplet/) and RecMotif (source code provided by the authors). iTriplet and RecMotif are both sample-driven algorithms which heuristically extract *q*-cliques from an *N*-partite graph (*q*=*N* for RecMotif because of the OOPS constraint). Meanwhile, we compared TFBSGroup with the pattern-driven algorithms SPELLER, WEEDER, and MITRA in order to reveal more differences between sample-driven and pattern-driven approaches. We then used TFBSGroup on a large data set of prokaryotic promoters generated from the *Escherichia coli* database RegulonDB for the purpose of finding real long and weak motifs. Also, we showed the results of TFBSGroup in discovering motifs in ChIP-seq data sets for 12 mouse transcription factors involved in ES cell pluripotency and self-renewal [28]. All experiments were performed on a computer with an Intel 2.99 GHz processor and 2GB of main memory running the Windows XP.

### Benchmark data sets

Like the previous work of Buhler and Tompa [9], the testing samples were generated synthetically using the following steps: 

1) A parent motif of length *l* was chosen by randomly picking *l* bases from the nucleotide set {*A*,*C*,*G*,*T*}.

2) *N* i.i.d. background sequences of length *L* were constructed at random.

3) *q* (*q*≤*N*) sequences were randomly selected from these *N* background sequences.

4) The following steps were performed for each of the selected *q* background sequences: 

4.1) An instance of the parent motif was created by randomly choosing *d* (*d*<*l*) positions of the motif and randomly mutating these *d* bases to one of the four nucleotides.

4.1) A consecutive substring of random length *l* was selected from each background sequence.

4.1) The substring in Step 4.2 was replaced with a newly generated instance of the motif.

In our experiments, unless otherwise specified, the number *N* and the length *L* of sequences in an (*l*, *d*) sample are set to 20 and 600, respectively.

### Comparisons between TFBSGroup and state-of-art algorithms using (*l*, *d*) samples

Firstly, to show the efficiency of TFBSGroup, we compared it with state-of-the-art algorithms including the pattern-driven SPELLER, WEEDER, and MITRA-count (MITRA for short) and the sample-driven iTriplet and RecMotif on the same testing samples (Table 1). Secondly, we tested the effect of the maximal length *L* of DNA sequences [20]. The test results are shown in Table 2. WEEDER(*q*) indicates the execution time of WEEDER given *q*, *q*/*f* indicates that WEEDER failed to find the true motif for the given value *q*, TFBSGroup(*x*) indicates the run time of TFBSGroup given *x* (*d*≤*x*≤2*d*), *s*, *m*, and *h* denote seconds, minutes, and hours respectively, and ^′^−^′^ indicates a run time of over 10*h*.

**Table 1:** **Comparisons of TFBSGroup with state-of-art algorithms on (*l*,*d*) samples**

**(l,d)**	**SPE-**	**WEEDER**	**MI-**	**iTri-**	**Rec-**	**TFBSGroup(*x*)-**	**VINE**	**sMCL**
	**LLER**	**(q)**	**TRA**	**plet**	**Motif**	***1/5/10/b10/f***		
(10, 2)	18.83*s*	3.7*s* (20)	1.98*s*	0.17*s*	0.64*s*	17.2*s*(3)	*	15*s*
						29/30/30/0/70		
(11, 2)	17.33*s*	3.47*s* (20)	2*s*	0.28*s*	0.13*s*	14*s*(3)-1	8*s*	*
						99/100/100/0/0		
(11, 3)	4.52*m*	20.02*s* (17)	22.95*s*	9.78*s*	1.56*m*	14*s*(3)-1	*	*
						76/86/87/6/7		
(12, 3)	4.5*m*	6.63*m* (16)	22.92*s*	4.89*s*	0.59*s*	15.8*s*(4)-1	7*s*	24*s*
						68/72/72/0/28		
(13, 3)	4.54*m*	2.77*m* (18)	22.73*s*	1.28*s*	0.22*s*	15.6*s*(4)-1	*	*
						97/100/100/0/0		
(14, 4)	1.04*h*	34.97*m* (19)	4.3*m*	1.09*m*	18.3*s*	17*s*(5)-1	*	1.63*m*
						88/94/94/1/5		
(15, 4)	1.04*h*	7.2*m* (19)	4.28*m*	5.79*s*	0.70*s*	15.9*s*(5)-1	5.6*m*	*
						95/98/99/0/1		
(15, 5)	-	4.09*h* (19)	43.86*m*	11.5*m*	-	18.8*s*(5)-1	6.8*m*	*
						70/94/94/0/6		
(16, 5)	-	4.2*h* (19/*f*)	44.53*m*	3.78*m*	24.34*m*	18*s*(6)-1	6.8*m*	4.22*m*
		8.19*h* (18)				96/100/100/0/0		
(17, 6)	-	-	7.0*h*	4.58*h*	-	18.6*s*(7)-1	7.5*m*	*
						81/90/92/2/6		
(18, 6)	-	1.39*h* (20/*f*)	7.46*h*	27.86*m*	-	19.2*s*(7)-1	*	10.53*m*
		4.26*h* (19)				94/100/100/0/0		
(19, 7)	-	-	-	-	-	19.6*s*(8)-1	8.3*m*	*
						83/100/100/0/0		
(21, 8)	-	-	-	-	-	21.4*s*(9)-2	10.6*m*	*
						90/100/100/0/0		
(23, 9)	-	-	-	-	-	20.2*s*(10)-1	12.1*m*	*
						86/100/100/0/0		
(24, 8)	-	-	-	-	-	21.3*s*(11)-1	*	*
						97/100/100/0/0		
(25, 10)	-	-	-	-	-	22.1*s*(12)-1	13.4*m*	*
						90/100/100/0/0		
(40, 14)	-	-	-	-	4.28*h*	27.2*s*(21)-1	*	*
						98/100/100/0/0		

**Table 2:** **The influence of sequence length*l* on (15, 4)**

***L***	**SPELLER**	**WEEDER(*q*)**	**MITRA**	**iTriplet**	**RecMotif**	**TFBSGroup(*x*)-**	**VINE**
						**1/5/10/b10/f**	
300	10.9*m*	23.45*s* (20)	1.46*m*	1.18*s*	0.08*s*	4.8*s*(6)-	*
						100/100/100/0/0	
400	23.13*m*	1.88*m* (19/*f*)	2.38*m*	1.02*s*	0.19*s*	8.1*s*(6)-	*
		3.55*m* (18)				100/100/100/0/0	
500	40.89*m*	3.54*m* (19)	3.33*m*	4.88*s*	0.36*s*	11.3*s*(6)-	*
						98/100/100/0/0	
600	1.05*h*	7.5*m* (19)	4.3*m*	4.11*s*	0.74*s*	16.1*s*(5)-	11.7*m*
						95/98/99/0/1	
700	1.48*h*	13.83*m* (19/*f*)	5.21*m*	9.77*s*	1.16*s*	21.8*s*(5)-	19.6*m*
		34.17*m* (18)				98/100/100/0/0	
800	2.03*h*	24.56*m* (19)	6.25*m*	16.53*s*	2.03*s*	28.5*s*(5)-	27.0*m*
						99/100/100/0/0	
900	2.78*h*	37.24*m* (19)	7.48*m*	43.92*s*	3.17*s*	36.5*s*(5)-	25.3*m*
						98/99/100/0/0	
1000	3.62*h*	51.47*m* (19/*f*)	8.8*m*	45.52*s*	4.86*s*	44.8*s*(5)-	43.5*m*
		1.67*h* (18)				99/100/100/0/0	

To demonstrate the accuracy of our algorithm, we ran TFBSgroup on 100 randomly generated test samples for each (*l*, *d*) pair and reported the number of samples in which the implanted motifs were correctly reported in the top 1/5/10, and which were correctly identified but listed below the top 10 (denoted *b*10). We also reported the number of samples in which the implanted motifs were not correctly reported by TFBSGroup (denoted *f*), since our algorithm TFBSGroup may fail in some cases. The accuracy of TFBSGroup for different (*l*, *d*) pairs is shown as 1/5/10/*b*10/*f* after TFBSGroup(*x*) in Tables 1 and 2. For example, we correctly found motifs ranked first in 95 samples, motifs ranked within the top 5 in 98 samples, and motifs ranked within the top 10 in 99 samples for (15, 4). However, we failed on one sample set. Thus, the accuracy of TFBSGroup on 100 (15, 4) samples can be estimated as 95/98/99/0/1, where motifs were ranked by their significance score [14,15]. Furthermore, since the run times of TFBSGroup on different samples of the same (*l*, *d*) pair showed negligible difference (usually <1 second) under the same parameter settings, we did not average the run times of 100 samples for an (*l*, *d*) pair but instead kept the run times of TFBSGroup on the same sample sets used in the efficiency comparisons with other algorithms.

In addition, Table 1 and Table 2 describe the results of VINE (Huang et al. [20]) and sMCL-WMR (Boucher and King [23], sMCL for short) in order to compare these sample-driven algorithms, which extract cliques from *N*-partite graphs, to our work. ^′^∗^′^ indicates that no result was available from literature. The experiments using VINE were performed on a PC with an Intel Pentium IV 3.40 GHz processor and 1GB of main memory running Windows. Those for sMCL-WMR were performed on a Linux machine with a 2.6 GHz processor and 1Gb of RAM running Ubuntu Linux. We also tested iTriplet and found that the run times of two implementations of this algorithm on the same (*l*, *d*) sample were greatly different due to its random mechanism for selecting two reference sequences and an *l*-mer within a reference sequence. Taking five runs of iTriplet on the same (15, 4) sample as an example, the minimum execution time was 0.859 seconds and the maximum was 9.156 seconds. We have reported the average run time of 5 runs of iTriplet.

As shown in Table 1, the sample-driven algorithms run faster than the pattern-driven variety. However, except for MITRA, we used only *q* and *d* as input in all implementations of the pattern-driven algorithms. The length *l* of planted motifs can be predicted by these algorithms while all the sample-driven types tested above and MITRA require *l*, *d* and *q* to be specified in advance. TFBSGroup can find all long and weak (*l*, *d*) motifs within 30 seconds under the ZOMOPS constraint. Most planted motifs in the synthetic samples (with the exception of (10, 2)) were correctly reported in the top 1/5/10. TFBSGroup performed with high accuracy when identifying exact matches for long and weak (*l*, *d*) motifs such as (15, 4), (16, 5), (18, 6), and (19, 7). However, TFBSGroup failed to find exact planted motifs in many cases involving conserved motifs (e.g., (10, 2)). This may be because the networks are too sparse to form good communities. For hard (*l*, *d*) motif search problems such as (15, 5), (16, 5), (17, 6), and (18, 6), TFBSGroup is much more efficient than iTriplet, RecMotif, VINE, and sMCL-WMR. In addition, it is not easy to tune the parameter *q* in WEEDER since, with the decrease of *q*, the run time is dramatically increased. Moreover, according to our experiments, iTriplet cannot be guaranteed to find true inserted motifs in all cases because of its random mechanism. Also, the memory usage of iTriplet is much higher and can freeze the computer during searches for long and weak motifs such as (19, 7).

Table 1 and Table 2 show that the sample-driven algorithms are more sensitive to the length *L* of DNA sequences, which influences the scale and the noise ratio of an *N*-partite graph given *x*. The pattern-driven approach is much more sensitive to *l* and *d*, which dominate the scale of the search space. These results are consistent with the time complexity of these algorithms collected from [21,22] and shown in Table 3. According to Table 1 and Table 2, the value of *q* has a strong influence on the efficiency of WEEDER. For instance, when *L*=700, the true motif could not be found when we set *q*=19. We then let *q*=18 and ran WEEDER again. The run time when *q*=18 was two to three times longer than when *q*=19. We also observed that a shorter length *L* of sequences led to a more accurate TFBSGroup result. This is consistent with the theoretical results in Zia and Moses [29].

**Table 3:** Algorithmic complexity

**Pattern-D**	**Time*O(·)***	**Space*O(·)***	**Sample-D**	**Time*O(·)***	**Space*O(·)***
SPELLER	*O*(*N*^2^*LN*(*l*,*d*))	*O*(*N*^2^*L*/*w*)	iTriplet	*O*(*NL*^3^*p*(*l*,2*d*)*l*^3^*d*^2^)	*O*(*N*(*l*,*d*))
WEEDER	*O*(*N*^2^*LN*(*l*,*d*))	*O*(*N*^2^*L*/*w*)	RecMotif	≤*O*(*NL*^7^*p*(*l*,2*d*)^10^)	*O*(*N*^2^*L*)
MITRA	*O*(*NLlN*(*l*,*d*))	*O*(*NLl*)	VINE	*O*(*N*^4^*L*^4^)	*O*(*N*^2^*L*^2^)
			TFBSGroup	*O*(*p*(*l*,*x*)^2^*N*^4^*L*^4^)	*O*(*p*(*l*,*x*)*N*^2^*L*^2^)

### Mining for transcription factor binding sites in *Escherichia coli* K-12

**Figure 1:**
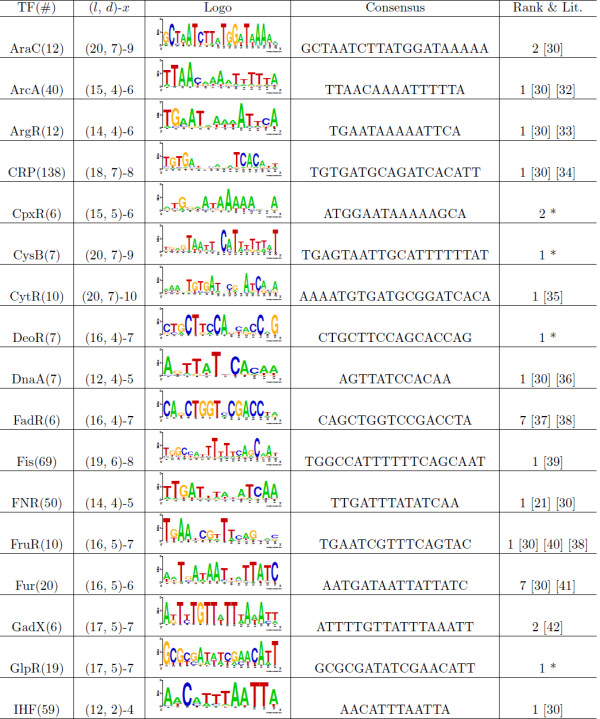
**Motifs in *Escherichia coli *K-12 promoter regions.** This figure shows the motifs predicted by TFBSGroup in the first part of *Escherichia coli* K-12 promoter regions ordered by alphabet [21,30,32-42].

**Figure 2:**
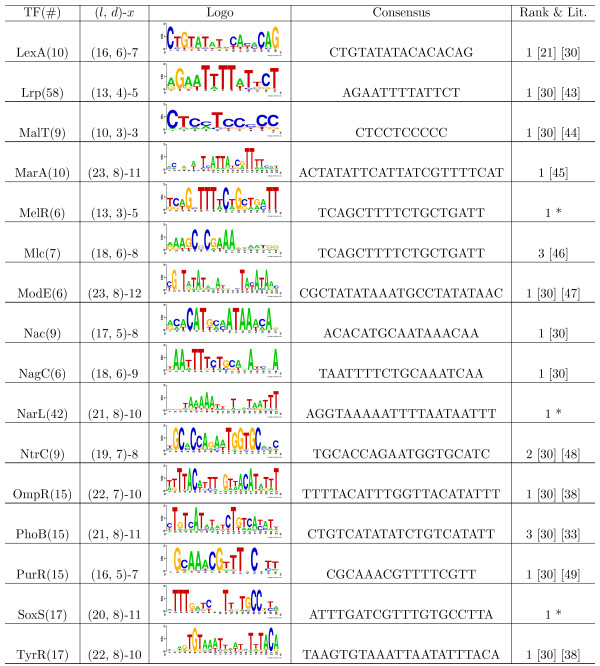
**Motifs in*Escherichia coli *K-12 promoter regions (cont’d).** This figure shows the motifs predicted by TFBSGroup in the remaining part of *Escherichia coli* K-12 promoter regions ordered by alphabet [21,30,33,38,43-49].

In order to further evaluate TFBSGroup, we used the algorithm on a large data set (ECRDB70-10) to find the binding sites within promoters of *Escherichia coli* K-12 DNA. This data, collected by Hu et al. [25], is stored in RegulonDB [27] and contains groups of sequences with experimentally-determined binding sites in the middle regions of the sequences. We selected sequence sets with more than five DNA sequences. We used published motif consensuses in the literature, especially the results in Li et al. [30], as a guide for inferring the values of *l* and *d*. We listed the motif consensuses, which were similar to the consensuses published in the literature. If no exact match or similar result was found in the literature, we listed the top ranked motif consensuses with the most binding locations in the middle regions of the sequences. The test results (obtained within 1 minute by running TFBSGroup) are shown in Figures 1 and 2, where ‘TF’ indicates the name of the transcription factor, ‘ *#*’ indicates the number of sequences in the corresponding set, ‘Consensus’ indicates the motif consensus of the corresponding TFBSs given a TF, ‘Logo’ indicates the sequence logo of all TFBSs for a specified TF (created using the web-based application tool Weblogo [31]), ‘Rank’ indicates the ranking number of the significance score [14,15] for the motif consensuses, ‘Lit.’ indicates that similar motif consensuses have been published in the literature while ‘*’ means no similar motif consensus was found in the literature, and (*l*,*d*) and *x* are represented in the same way as in Table 1.

As shown Figures 1 and 2, TFBSGroup can efficiently find over-represented long motifs from prokaryotic promoters. We illustrate this point using the well-studied TFs CRP, FNR, and LexA as examples [21]. Binding site data for the CRP protein includes 138 DNA sequences of length 219 with the consensus TGTGAnnnnnnTCACA (consensus model: (18, 7)) and 138 actual binding sites. The FNR data includes 50 DNA sequences of length 222 with the consensus TTGATnnnnATCAA (consensus model: (14, 4)) and 50 actual binding sites. The LexA data includes 10 DNA sequences of length 222 with the consensus CTGTnnnnnnnnnnCAG (consensus model: (16, 6)) and 10 actual binding sites. For all three sets, the expected motifs are ranked first by TFBSGroup in terms of their significance score: CRP is reported to have 121 sites (63 true), FNR is predicted to have 46 sites (23 true), and LexA is reported to have 12 sites (8 true). The *precision* on the site level ($\text{precision}=\frac{\text{TP}}{\text{TP}+\text{FP}}$) is close to or greater than 50% on these three data sets, where *TP* is the number of true positive sites and *FP* is the number of false positive sites. It should be pointed out, however, that some results marked with an ‘*’ in Figures 1 and 2 may not be satisfactory due to the low specificity of binding sites for the TFs, insufficient number of sequences from which to a draw statistical conclusion, or a lack of knowledge of the proper (*l*, *d*) models. Compounding the problem is the fact that the true consensuses in these data sets are unknown, a difficulty which exists for all consensus model-based algorithms.

### Motif discovery in 12 mouse ES CELL ChIP-seq data sets

**Figure 3:**
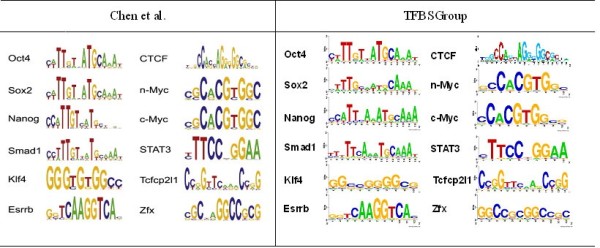
**Motifs in 12 mouse ES CELL ChIP-seq data sets.** This figure shows the motifs predicted by TFBSGroup in 12 mouse ES CELL ChIP-seq data sets and compares these motifs with those identified in Chen et al. [28].

**Figure 4:**
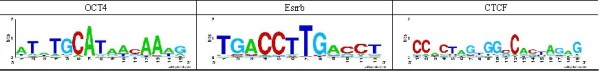
**Alternative motifs for OCT4, Esrrb, and CTCF.** This figure shows alterative motifs predicted by TFBSGroup for TFs OCT4, Esrrb and CTCF.

To further evaluate the accuracy of motifs predicted by TFBSGroup, we analyzed the ChIP-seq data sets for 12 DNA-binding TFs (CTCF, cMyc, Esrrb, Klf4, Nanog, nMyc, Oct4, Smad1, Sox2, STAT3, Tcfcp2I1, and Zfx) involved in mouse embryonic stem cell pluripotency and self-renewal [28]. To prepare the data sets for use with motif discovery algorithms, we first extracted peak regions from ChIP-seq data using MACS [50] with a FDR (false discovery rate) threshold of 0.2. We then mapped the centers of the ChIP-seq peaks to the mouse mm10 assembly and extracted 100-bp of genomic sequence centered around each peak. To compare motifs identified by TFBSGroup with motifs found in Chen et al. [28], we ran TFBSGroup on hundreds of peaks with low *p*-values. The results of Chen et al. and TFBSGroup are shown in Figure 3, where all sequence logos predicted by TFBSGroup, including those in Figure 4, were also created using the web-based application tool Weblogo [31]. We found motifs matching those identified in Chen et al. [28]. Specifically, motif logos predicted by Chen et al. [28] and TFBSGroup for each TF in Figure 3 (with the exception of Klf4 and Zfx) are exactly or ‘almost’ exactly the same. However, it is well known that Klf4 is able to recognize GC-rich regions. ZFX has no known published consensus sequence, but its predicted motif agrees to some extent with the result of Chen et al. and the result predicted by cEMRMIT [51].

In [28], Chen et al. used WEEDER and then refined and extended the motifs with an Expectation-Maximization method. This second step was necessary because the supplied version of the WEEDER algorithm limited the motif search to a maximum of 12 bps. As discussed in the previous sections, WEEDER operated with low efficiency for long motifs and was difficult to tune for the parameter *q*. On the contrary, TFBSGroup was able to find both long and weak motifs. We obtained the motifs and their TFBS locations in sequences within 1 minute for all data sets with just one run of TFBSGroup.

In addition, we found alternative motifs for some TFs such as OCT4, Esrrb and CTCF, which were also reported in a previous study [52]. One significant alternative motif for each of the three TFs is shown in Figure 4. The TFBS sequences of this alternative motif were complementary to those of the main motif in Figure 3 for each of three TFs.

## Conclusions and discussion

In this work, we have developed a novel and efficient algorithm (TFBSGroup) for identifying (*l*, *d*) motifs under the ZOMOP constraint. It extracts dense subgraphs from an *N*-partite graph using a fast community detection algorithm designed for processing large-scale networks (BGLL). Based on these extracted communities, TFBSGroup heuristically refines candidate motifs and their instances towards the true motifs. Experimental tests on synthetical samples have shown that TFBSGroup can very quickly discover long and weak (*l*, *d*) motifs and their instances. More importantly, TFBSGroup has achieved good performance in rapidly identifying motifs in a large data set of promoters generated from *Escherichia coli* and in accurately discovering motifs in ChIP-seq data sets for 12 mouse transcription factors involved in ES cell pluripotency and self-renewal. Still, TFBSgroup may not work well in the extreme case that the number of mutations between each motif instance and the motif itself is exactly *d*, since the graph will be too dense to be partitioned sufficiently. Fortunately, this case seldom occurs in real applications. In the future, we plan to improve the algorithm by combining structure- and sequence-based methods in order to address this issue. Also, we plan to improve the algorithm’s ability to process large-scale ChIP-Seq data sets.

## Methods

### (*l*, *d*) motif search and dense subgraph extraction

Given a set of sequences *S*={*s*_1_,*s*_2_,…,*s*_*N*_} over a symbol set *Σ*={A,C,G,T} and positive integers *l* and *d* (|*s*_*i*_|≤*L*, 1≤*i*≤*N*, 1≤*l*≤*L* and 0≤*d*<*l*), an (*l*,*d*) motif search finds a string *t*∈*Σ*^*l*^ such that for at least *q* (*q*≤*N*) sequences {*s*_*i*1_,*s*_*i*2_,⋯,*s*_*iq*_}⊆*S* there exists a substring *t*_*ij*_ in each *s*_*ij*_ (*j*=1,2,⋯,*q*) with *d*(*t*,*t*_*ij*_)≤*d*, where *d*(*t*,*t*_*ij*_) indicates the Hamming distance between the two strings *t* and *t*_*ij*_.

Since the Hamming distance between each instance of a motif and the motif itself is at most *d*, the Hamming distance between two instances is no more than 2*d* and all instances of the true motif must form a *q*-clique. Therefore, we can obtain (*l*, *d*) motifs by extracting *q*-cliques from an *N*-partite graph where each vertex is an *l*-mer in *S* and there is an edge between two *l*-mers *l*_*i*_ and *l*_*j*_ (*l*_*i*_ and *l*_*j*_ are *l*-mers of *s*_*i*_ and *s*_*j*_, respectively, *i*≠*j*) if the Hamming distance between the two *l*-mers is no more than 2*d*.

As far as synthetic samples randomly generated by the method mentioned in the above section are concerned, the probability of two random *l*-mers with a maximum distance of *x* is

(1) $$p_{\left(l,x\right)}=\overset{x}{\underset{i=0}{\sum }}\left(\begin{array}{l} i\\ l \end{array}\right){\frac{3}{4}}^{i}\cdot {\frac{1}{4}}^{\left(l-i\right)}.$$

Thus, for a set of *N* sequences with length *L*, there are 0.5×*N*×(*L*−*l*+1)×(*N*−1)×(*L*−*l*+1)×*p*_(*l*,2*d*)_ random edges in the background sequences. For example, there are an estimated 18.2 million random edges in the background sequences for an (18, 6) sample when *N*=20 and *L*=600. There are also some spurious edges around the vertices of motif instances, especially for long and weak (*l*, *d*) motifs, since the neighbor vertices of a motif instance may have links to the neighbor vertices of other motif instances. Still, only *q*∗(*q*−1)/2≤*N*∗(*N*−1)/2 edges are true positive links forming an expected *q*-clique.

Suppose there is an edge between two vertices in an *N*-partite graph if the Hamming distance of the vertices is no more than *x* (*d*≤*x*≤2*d*) instead of 2*d*. In this case, the number of spurious edges may be dramatically reduced. For example, if we set *x*=7 for a real (18, 6) sample, there are only 82,343 edges in the *N*-partite graph (there are an estimated 80,335 edges using Eq. 1). However, the instances of a motif in this situation may not form a *q*-clique but rather a densely connected subgraph. We can obtain an (*l*, *d*) motif by detecting dense subgraphs in an *N*-partite graph where the distance of two vertices is at most *x*.

### Community detection and dense subgraph identification

In recent years, complex network analysis has been highlighted in the research community. It is a powerful tool used to describe the structure of many complex systems in nature and society and has many potential applications. A network is usually represented by a graph *G*=(*V*,*E*), where *V* is a set of *n* vertices and *E* is a set of *m* edges representing relationships between pairs of vertices.

Community structure is one of the most important topological characteristics in a network. A community structure is a subgraph of a network whose vertices are more highly connected with each other than with vertices outside the subgraph. Therefore, the problem of community detection requires the partition of a network into communities of densely connected nodes such that ∀*u*,*v*,*u*≠*v*,*C*_*u*_∩*C*_*v*_=*∅* and ∪_*u*_*C*_*u*_=*V*, where *C*_*u*_ (or *C*_*v*_) is one of the partitioned communities. It should be apparent that community structure is a type of dense subgraph. The current algorithms for identifying communities in complex networks can be used to find dense subgraphs within graphs. Many methods to identify community structures in complex networks have been developed [53,54]. As mentioned above, however, an *N*-partite graph of input sequences with a long and weak (*l*, *d*) motif may be a large network with millions of edges. Fast community detection methods are required to partition a large-scale graph. In the field of complex network analysis, algorithms including Infomap [55], BGLL [26], LPA [56], and RG [57] are designed to efficiently detect communities in very large networks. In this study, we use the BGLL algorithm [26] to find dense subgraphs in an *N*-partite graph. This algorithm is best for our purposes since we only need a coarse partition and BGLL is very fast and easy to use. The source code for this software can be obtained from http://findcommunities.googlepages.com/.

### BGLL: a near linear time algorithm for community detection

BGLL is a heuristic method for optimizing modularity (Eq. 2), which measures the difference between the empirical distribution of in-community connections of a partition and the expected distribution of in-community connections of a partition in a randomly generated graph with the same vertex degree distribution [58].

(2) $$Q=\frac{1}{2m}\underset{i,j\in V}{\sum }[A_{\text{ij}}-\frac{k_{i}k_{j}}{2m}]\delta (C_{i},C_{j}),$$

where *A*_*ij*_ is the weight of the edge (*i*,*j*). If a network is unweighted, *A*_*ij*_=1 for (*i*,*j*)∈*E*, otherwise *A*_*ij*_=0. $k_{i}=\overset{n}{\underset{j=1}{\sum }}A_{\text{ij}}$ is the sum of the weights of the edges attached to vertex *i* or the degree of the node *i* for an unweighted network. *C*_*i*_ is the community to which the node *i* is assigned and *δ*(·,·) is the *Kronecker* function. A larger *Q* yields a better partition.

The BGLL algorithm can be divided into two iterative phases. Firstly, it assigns a different community to each node of a network. Then, for each node *i*, it considers the neighbors *j* of *i* ((*i*,*j*)∈*E*) and evaluates the gain of modularity *ΔQ* (Eq. 3) that would take place by removing *i* from its community and placing it in the community of *j*.

(3) $$\begin{array}{l} \mathrm{\Delta Q}=\left[\frac{\underset{\text{in}}{\sum }+2k_{i,\text{in}}}{2m}-{\left(\frac{\underset{\text{tot}}{\sum }+k_{i}}{2m}\right)}^{2}\right]\\ \;-\;\left[\frac{\underset{\text{in}}{\sum }}{2m}-{\left(\frac{\underset{\text{tot}}{\sum }}{2m}\right)}^{2}-{\left(\frac{k_{i}}{2m}\right)}^{2}\right], \end{array}$$

where $\underset{\text{in}}{\sum }$ is the sum of the weights of the edges inside a community *C*_*u*_, $\underset{\text{tot}}{\sum }$ is the sum of the weights of the edges incident to nodes in *C*_*u*_, and *k*_*i*,*in*_ is the sum of the weights of the edges from *i* to nodes in *C*_*u*_. If the gain is negative, *i* stays in its original community, otherwise, *i* is placed in the community which provides maximum gain. The second phase of the algorithm involves building a new network whose nodes make up the communities found during the first phase. The weights of the edges between the new nodes are the sum of the weight of the edges between nodes in the corresponding two communities. Edges between nodes of the same community lead to self-loops for this community in the new network. The algorithm naturally incorporates a notion of hierarchy, which results in communities of communities.

The BGLL algorithm is extremely fast and performs in linear time on typical and sparse data, since the gain of modularity is very easy to compute with Eq. 2 and the number of communities decreases drastically after just a few passes. Most of the run time lies within the first iteration [26]. In this study, we took the results of the first iteration only since we are interested in obtaining coarse-grained candidate motifs and their group of instances from dense subgraphs of the original network and are not concerned about the hierarchical structure of communities.

### TFBSGroup: a fast motif-finding algorithm

TFBSgroup operates in two phases. Firstly, we construct an *N*-partite graph where the distances of pairs of vertices are no more than *x* (*d* ≤ *x* ≤ 2*d*) for a set of input sequences, which is assumed to contain an (*l*, *d*) motif and at least *q* (*q*≤*N*) instances. We then detect all communities with a size of at least *t* (the default is *q*/2) in the *N*-partite graph using the BGLL algorithm to obtain a candidate motif consensus by aligning all *l*-mers in each community. Secondly, we greedily refine these candidate motifs toward the true motif using the following three steps: 1) For each candidate motif consensus, we look for the vertices in the neighbor set of the current community *C*_*u*_ for which the Hamming distance between the consensus and the corresponding *l*-mers of these vertices are no more than *d* in order to form a new community. A vertex belonging to the new community is in the neighbor set of the current community *C*_*u*_ if the position of the corresponding *l*-mer of the vertex is in the interval $\left[\text{max}\{0,\text{po}s_{v_{i}}-l\},\text{min}\{\text{po}s_{v_{i}}+l,L-l\}\right]$. $\text{po}s_{v_{i}}$ is the start position of the corresponding *l*-mer of a vertex *v*_*i*_ (*v*_*i*_∈*C*_*u*_) in the sequence to which it belongs. 2) We align the new community to obtain a new candidate motif consensus. We then iteratively execute step 1 and step 2 until each new candidate consensus cannot be changed. 3) We shift the corresponding *l*-mers (an *l*-mer corresponds to a vertex *v*_*i*_ in the final community) of the final candidate motif in the interval $\left[\text{max}\{0,\text{po}s_{v_{i}}-\lfloor l/3\rfloor \},\text{min}\{\text{po}s_{v_{i}}+\lfloor l/3\rfloor ,L-l\}\right]$, since the true motif and its instances may be near the final candidate motif consensus and their instances.

Furthermore, since there may be many false positive motifs, we sort all output motifs according to their statistical significance using the method of Pavesi et al. [14,15] and then delete the duplicates. Finally, the top *k* significant motifs and their instances are returned. TFBSGroup is so named because it completes its run after all potential motifs and the groups of their instances (corresponding to the groups of TFBSs in a set of DNA sequences) are reported as output.

The details of the TFBSGroup algorithm are shown below, where (*i*, *j*) indicates an instance starting at the *j*-th position of the *i*-th sequence *s*_*i*_. *t* (default = *q*/2) is used to filter false positive groups forming small communities during the initial stage. This will not affect the result or the speed of the algorithm in our simulations because the largest group usually has the highest significance score. We can set *t*=0 to ensure all candidate groups are examined. Furthermore, the window size ⌊*l*/3⌋ is used to ensure that the predicted TFBSs are within its inserted positions and not around them. Generally, we can identify true motifs when the window size is less than *l*/3. The source code for TFBSGroup can be obtained from http://bioinformatics.bioengr.uic.edu/TFBSGroup/ or Additional file 1.

**Algorithm 1:** The TFBSGroup Algorithm

### Time and space complexity of TFBSGroup

The time complexity of TFBSGroup depends mainly on the first phase of the algorithm, which includes time for constructing an *N*-partite graph with distance *x* (*d*≤*x*≤2*d*) and time for extracting communities from the constructed *N*-partite graph. During the second phase, the algorithm searches through candidate motif consensuses and their instances within each of the communities.

There are at most *N*×(*L*−*l*+1)*l*-mers for a set of DNA sequences with a length of at most *L*. Therefore, there are at most *N*×(*L*−*l*+1)×(*N*−1)×(*L*−*l*+1)×*l*/2=*O*(*N*^2^×*L*^2^×*l*) comparisons for constructing an *N*-partite graph. During one pass of BGLL, the algorithm computes *ΔQ* at most *t* times for each vertex in a network, where *t* is the maximum number of neighbors of a vertex in the network. The time complexity of BGLL is bound by *O*(*N*×*L*×*t*×*time*_*ΔQ*_) for extracting communities from an *N*-partite graph because there are at most *N*×(*L*−*l*+1) vertices in a network, where *time*_*ΔQ*_ is the time complexity for computing *ΔQ*. As a result, the time complexity of TFBSGroup for the worst case is

$$\begin{array}{lll} O(t\times N\times L\times \text{tim}e_{\mathrm{\Delta Q}}) & =O(t\times N\times L\times m)\; & \;\\ & ≅O(p{\left(l,x\right)}^{2}\times N^{4}\times L^{4}),\; & \; \end{array}$$

where *m* is the number of edges in a network, *m*=*O*(*p*(*l*,*x*) × *N*^2^ × *L*^2^) and *t*=*O*(*p*(*l*,*x*) × *N* × *L*), as estimated using Eq. 1. However, it should be noted that the above bound can be a substantial overestimate. The time complexity of TFBSGroup is almost equal to the time complexity of BGLL, which is near linear with respect to *m* in real applications, especially for sparse networks [26].

The space complexity of TFBSGroup is mainly affected by the storage of all *l*-mers and an *N*-partite graph, where the distance of two vertices is at most *x*. Thus, the space complexity of TFBSGroup is *O*(*m*)=*O*(*p*(*l*,*x*)×*N*^2^×*L*^2^), while it is at least *O*(*p*(*l*,2*d*)×*N*^2^×*L*^2^) for previous graph-based algorithms.

The time and space complexity of TFBSGroup and several related algorithms (except for sMCL, since no complexity analysis is available for this algorithm in the literature [23]) are shown in Table 3, where the left half lists pattern-driven algorithms (labeled ‘Pattern-D’) and the right half lists sample-driven algorithms (labeled ‘Sample-D’). $N(l,d)=\overset{d}{\underset{i=0}{\sum }}\left(\begin{array}{l} i\\ l \end{array}\right)3^{i}$and *w* is the word length, which corresponds to bit length of a processor. The time complexity of RecMotif relies heavily on the value of *p*(*l*,2*d*) (see Table one in Sun et al. [22]). According to Table 3, each algorithm has its own advantages. The time complexity of pattern-driven algorithms is higher but they have lower space complexity. The time complexity of sample-driven algorithms is lower but they generally have higher space complexity. RecMotif is too sensitive to *p*(*l*,2*d*) and *L*. When *p*(*l*,2*d*) is small, RecMotif runs very fast. However, when *p*(*l*,2*d*) is larger than 0.28, RecMotif can not produce the results within a reasonable amount of time. TFBSGroup is a complement to sample-driven algorithms since it makes a reasonable trade-off between speed and accuracy.

### The choice of *x*

**Figure 5:**
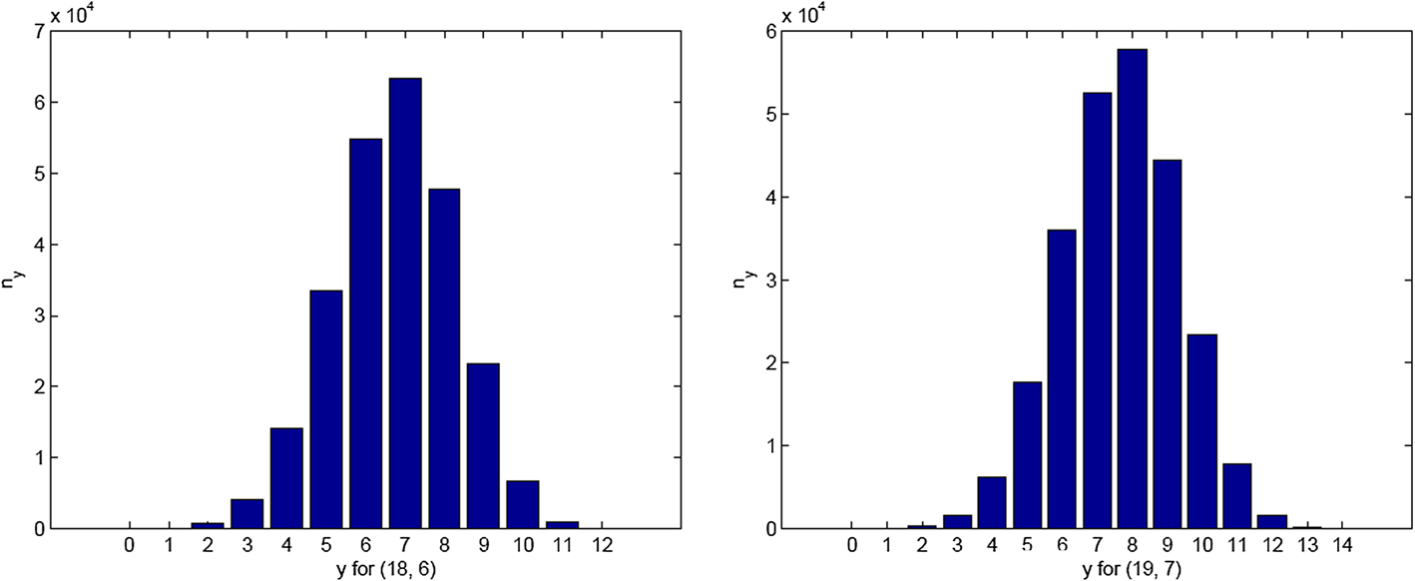
**The histogram of*n***_***y***_** for (18, 6) and (19, 7).** The height of a panel in the figure correspond to the number ***n***_***y***_ of instance pairs with distance *y* (*y*={0,1,2,⋯,2*d*}).

The key problem with TFBSGroup is the selection of the parameter *x*. If *x* is too large, the *N*-partite graph may be too dense to define a community containing only the instances of a motif. If *x* is too small, the graph is too sparse to form the expected communities and the true group of TFBSs will be missed. In this study, we use an experimental statistical method to estimate *x* for a specified (*l*, *d*) motif search problem. Firstly, for a given *l*-mer consensus, we created 500 instances of the consensus with the Hamming distance between the consensus and each instance equal to at most *d*. We then computed the Hamming distance for each pair of instances and counted the number *n*_*y*_ of instance pairs with distance *y* (*y*={0,1,2,⋯,2*d*}) to get the frequency *n*_*y*_ distribution. The center of this distribution should be an estimation of *x*, i.e., *x*≥*max*{*n*_*y*_,*y*∈{0,1,2,⋯,2*d*}} or close to this. Taking (18, 6) and (19, 7) as examples, the histograms of the frequency *n*_*y*_ distribution are shown in Figure 5. Using these distributions as a guide, we set *x*=7 and *x*=8 for (18, 6) and (19, 7), respectively.

## Competing interests

The authors declare that they have no competing interests.

## Authors’ contributions

CJ initiated the project, analyzed the data, and drafted the manuscript; MC and JY participated in the study design, discussion, and editing of the manuscript. All authors read and approved the final manuscript.

## Supplementary Material

Additional file 1 The C++ Version of TFBSGroup (for WindowsXP). Click here for file
